# Reliability and construct validity of a composite pain scale for rabbit (CANCRS) in a clinical environment

**DOI:** 10.1371/journal.pone.0221377

**Published:** 2020-04-30

**Authors:** Penelope Banchi, Giuseppe Quaranta, Alessandro Ricci, Mitzy Mauthe von Degerfeld

**Affiliations:** 1 C.A.N.C. (Centro Animali Non Convenzionali), Dip. di Scienze Veterinarie, Università degli Studi di Torino, Grugliasco, Turin, Italy; 2 Dip. di Scienze Veterinarie, Università degli Studi di Torino, Grugliasco, Turin, Italy; University of Lincoln, UNITED KINGDOM

## Abstract

A composite pain scale for assessing and quantifying pain in rabbits (CANCRS) has been designed merging the Rabbit Grimace Scale (RbtGS) and a scale including clinical parameters (CPS). Construct validity and inter-rater reliability were assessed for CANCRS, for RbtGS and for CPS, in order to test their potential to detect pain in a clinical setting. Rabbits (n = 116) were either hybrids or purebreds and they were independently evaluated by two raters, who could be veterinarians (V) or veterinary medicine students (S). Score intervals determined four pain classes (No pain, Discomfort, Moderate pain and Severe pain) that matched presumptive pain classes associated with some pathological conditions. A chi-square test was used to assess the construct validity of the scales by checking how frequently scale results and presumptive pain classes matched. Sixty-nine patients were evaluated by one V and one S, whereas forty-seven rabbits were assessed by two V, in order to test inter-rater reliability. An intra-class correlation coefficient (ICC) was used to test reliability of the scales, whereas Cohen’s kappa tested inter-rater agreement for each parameter of the CANCRS. Construct validity results show that CANCRS and RbtGS efficiently reveal pain (P ≤ 0.05), while CPS does not (p > 0.05). Inter-rater reliability was very good for both CANCRS and CPS (ICC 0.88 V-V, 0.94 between V-S; ICC 0.97 V-V, 0.91 V-S) and good for RbtGS (ICC 0.77 V-V, 0.88 V-S); therefore, CPS reproducibility was better between veterinarians and students than between veterinarians. Inter-rater agreement between veterinarians and veterinary medicine students was moderate to very good for all the parameters included in the CANCRS (Cohen’s kappa >0,60). In conclusion, it is possible to state that the CANCRS has construct validity and it is a reliable tool for use in clinical practice, when coping with many rabbits with morphological differences. It is easy and fast to use and enriches the RbtGS with some clinical parameters that should be monitored during any clinical examination, allowing for capture of the multidimensional aspect of pain.

## Introduction

Appropriate pain recognition and treatment represents an ethical obligation for veterinarians, in order to preserve the patient’s health and quality of life [1]. However, assessment and quantification of pain are arduous, due to great variability in pain expressions among individuals and species [2].

In rabbits, pain recognition can be particularly challenging because, as with many other prey species, these animals are predisposed to mask any sign of pain [3].

A recent review article about rabbit analgesia [3] describes how the percentage of veterinarians who doubt their knowledge on small mammals’ pain assessment (60%) is higher than that for dogs and cats (42% and 30% respectively) and the lack of a ‘gold standard’ method for pain assessment leads to difficulties in pain management.

To verify properly the effectiveness of analgesic drugs, a validated assessment tool is needed. Moreover, the drugs used to treat pain in rabbits, were tested on small numbers of animals, and few papers were published [3,4].

In rabbits, there is evidence that pain causes a decrease in activity and appetite [5,6]; since rabbits’ metabolism is geared to a constant supply of nutrients from the digestive tract, a decreased or absent food intake and the subsequent mobilization of fat reserves, can lead to ketoacidosis and hepatic lipidosis [7]. Therefore, providing analgesia in suffering patients is one of the reasons why pain assessment is essential [8,9].

Physiological parameters have a limited function in pain recognition as they can be altered in any stressful situation. An increase in heart and respiratory rate can be seen whenever the patient is handled [3,7,10], as it happens during clinical examinations.

Many behavioral changes are effective for assessing and quantifying pain [11,12], but sometimes a prolonged monitoring is required. Freezing [3] is the main behavioral feature presenting when rabbits respond to pain and distress by remaining motionless [4,13], especially in the presence of an observer.

Composite pain scales are available for many species, such as canines and felines [11,12,14] and represent a useful tool to conduct an immediate and structured pain quantification. Composite pain scales encourage the observer to consider some behavioral and physiological changes, that would not otherwise be considered, but their importance is undeniable when considering pain as a multidimensional experience [2]. However, there is no validated equivalent for rabbits and the only existing pain scale for this species is the Rabbit Grimace Scale (RbtGS) [15], which uses changes in facial expression to quantify pain. Grimace scales have been developed for several species, such as horses [16], sheep [17], ferrets [18] and small laboratory rodents [19,20].

The Rabbit Grimace Scale is based on five facial action units (FAU). The main limitations of RbtGS is that it was tested on New Zealand White (NZW) rabbits only and its performances were verified in standardized conditions. Furthermore, classes for pain grading and quantification have never been defined, therefore, the scale is not useful to determine whether patients need analgesic treatment. For these reasons, the RbtGS has not yet been proven to be suitable for assessing pain in clinical settings [3].

The objective of the current study was to develop and test a composite pain scale for the assessment and quantification of pain in rabbits (CANCRS) in a clinical environment, on different rabbit breeds. This composite pain scale combines the RbtGS with some clinical parameters (CPS). CANCRS, RbtGS and CPS were also tested independently. Another aim was to establish some preliminary score ranges for each scale, defining four pain classes and improve the identification and quantification of pain.

## Materials and methods

### Animals

In accordance with the Dipartimento di Science Veterinarie of Università degli Studi di Torino policy, this research does not require the approval of the ethical committee, for the utilization of our assessment tool does not modify the clinical process designed for each patient and rabbits were manipulated for routine diagnostic purposes only. Proper informed consent was collected from the owner prior to each clinical evaluation.

One hundred and sixteen client-owned rabbits admitted to the C.A.N.C. (the exotics and wild animals Veterinary Teaching Hospital of the Veterinary Science DPT. of University of Torino), were considered for inclusion, between the years 2016 and 2018. No restrictions were placed on the breed, sex, age or weight of the rabbits.

Patients were both healthy rabbits admitted for vaccination, checkups or neutering surgery and animals suffering from assorted conditions, such as dental diseases, traumatic injuries, dermatitis, gastric stasis, endoparasites infestation, urinary diseases, and abscesses.

Critically ill patients with respiratory distress syndrome in need of oxygen administration, were excluded from the study in order to avoid further stress to the animals. In some patients, facial expression was not evaluable (e.g. rabbits requiring nasogastric tube or Elizabethan collar), therefore they were excluded from the study as well. Stuporous and comatose states also represented exclusion criteria, considering stupor as a state of lethargy and immobility with diminished responsiveness to stimulation and coma as a deep state of prolonged unconsciousness and unresponsiveness to external stimuli [21].

Pain was always scored at admission, during the patient’s first clinical examination, prior to any potential analgesia administration or surgery. Since clinical examination always happened in the same room, environmental conditions, including noise and light conditions, where similar. When facial action units belonging to the RbtGS were assessed, rabbits were kept in a carrier; the patients were then taken out from the carrier and handled for clinical assessment.

### Pain scales

A multidimensional composite pain scale (CANCRS) was developed for rabbits, merging the RbtGS with a Clinical Parameters Scale (CPS), which includes some physiologic parameters (pupil dilation, respiratory rate, respiratory pattern, heart rate) and behavioral responses (response to palpation, mental status and vocalization). The choice of the CPS parameters were based on other pain scales developed for other species of mammals [14,22], bearing in mind that dealing with rabbits allows the clinician to monitor parameters that are less stressful for the patient. Furthermore, respiratory pattern was included, in order to have a more accurate overview of the respiratory response to pain, since polypnea is not pain specific. Finally, pupil dilation can occur in painful situations [23,24].

The RbtGS considers five facial action units (FAU): orbital tightening, cheek flattening, nostril shape, whisker position, and ear position. Each FAU was scored according to whether it was not present (score 0), moderately present (score 1) and obviously present (score 2). Lop eared rabbits’ ear morphology does not permit for evaluation of ear position properly, therefore this parameter was not considered when assessing pain in such breeds.

For the CPS, the scores were given as follow. In response to noxious stimulation, pupillary dilation reflex occurs [24]. No pupillometer was available and raters were asked to make a subjective assessment of whether the rabbits’ pupils were dilated (score 1) or not (score 0).

Since heart rate is extremely variable and difficult to assess in rabbits [7], raters were asked to score percentage increases of the maximum physiological value (250 bpm) [25]. Increases greater than 20% (score 1) and 50% (score 2) were considered, for increases smaller than 20% the score assigned was 0. Heart rate was measured by both raters with a stethoscope for 15 seconds and the result was multiplied by four, in order to obtain beats per minute.

The average respiratory rate for a healthy subject was considered in a values range from 30 to 60 bpm [25]; four possible scores assigned: rates of 60 bpm or less (score 0), rates between 61 and 72 (score 1), rates between 73 and 90 (score 2) and rates higher than 90 bpm (score 3).

Respiratory pattern was considered as eupneic (score 0) or dyspneic (score 1). Dyspnea is associated with a number of pulmonary and cardiac diseases, as well as diseases of the chest wall and anxiety. Respiratory pattern was included to provide a more complete description of the changes which the respiratory rate goes through during pain, as in human medicine, association between pain and dyspnea has been reported [26].

When the suspected painful area was delicately palpated by each rater, the reaction was recorded and classified as no reaction (score 0), reaction during the palpation (score 1) and reaction before the palpation (score 2).

For vocalizations, the possibilities were absence of vocalization (score 0), vocalization when touched (score 1), intermittent vocalization without any contact with the operator (score 2) or continuous vocalization (score 3).

The mental status was classified as ‘normal’ (score 0), ‘depression’ (score 1) and ‘obtundation’ (score 2); the state in which the patient has a reduced interest in the environment, but still responds to external stimuli, was described as ‘depression’; obtundation occurs when responsiveness to visual and auditory stimuli (e.g. hand clapping and fingers snapping) decreases and the patient tends to sleep more than normal with drowsiness in between sleep states [27].

The medical history of the patient was reported on each clinical form in order to establish a presumptive pain (PP) class according to literature [2], since it is plausible that pain is directly proportional to the damage extent, as occurs in humans [2]; No pain (NP), discomfort (D), moderate pain (MP) and severe pain (SP) were the four pain classes created. Table 1 summarizes presumptive levels of pain associated with illness or injuries [2,28] adjusted for rabbit medicine.

**Table 1:** Presumptive pain classes associated with some clinical conditions affecting rabbit patients [2].

No pain	Healthy patient presented for vaccine administration, regular checkups and prior to neutering surgery.
Discomfort	Minor trauma, small wounds, intestinal or bladder repletion, oral cavity minor lesions, or ectoparasites infestation.
Moderate Pain	Gastric stasis with moderate visceral distension, dental diseases, traumatic injuries of the skin, localized dermatitis, otitis externa, endoparasites infestation, cystitis, abscesses.
Severe Pain	Osteoarthritis, peritonitis, organomegaly, ocular disorders (ulcers, glaucoma, uveitis), tumors, torsion or distension of the gastrointestinal tract, urethral obstruction, thrombosis or ischemia, otitis media or interna, severe intestinal distension, inflammation, burning or ulcers involving a large area of the body, multiple and/or exposed fractures.

Raters were asked to report the time they spent on the evaluation of each rabbit on the printed copy of the scale, in order to establish the average time needed to use the assessment tool. Parameters of the three scales are summarized in Table 2.

**Table 2:** Parameters of the three scales.

**CANCRS**	RbtGS [16]	Orbital tightening (0–2)
		Cheek flattening (0–2)
		Nostril shape (0–2)
		Whisker position (0–2)
		Ear position (0–2)
	CPS	Pupil dilation (0–1)
		Heart rate percentage increases—based on 250 beats/min (0–2)
		Respiratory rate—based on 60 breaths/min (0–3)
		Respiratory pattern (0–1)
		Palpation of the painful area (0–2)
		Mental status (0–2)
		Vocalization (0–3)

CANCRS includes the five Facial Action Units (FAU) of the RbtGS [15] and some clinical parameters gathered in the CPS. For each parameter, two to four scores were possible. The final score is the total sum of the ones given to each parameter.

### Scoring method

Each of the 116 rabbits included in the study was evaluated independently by two raters during the veterinary clinical examination; therefore, the total number of assessments was 232. The raters could be either veterinarians (V) or a veterinarian and a veterinary medicine student (S). Two veterinarians and four students took part in the study.

Raters were familiar with using the scale and provided with a printed copy for each rabbit. To ensure consistency, each rater also received clear instructions on how to use the scale and they were supported by the presence of images explaining how to evaluate the FAU considered by the RbtGS. Rabbits were scored during the first clinical examination and raters were instructed to first fill in the RbtGS part, assessing the rabbit visually from a distance, prior to opening the same carrier in which the rabbit was admitted to the hospital, in order to avoid further stress. Afterwards, raters had to interact with the patient and fill in the second part (CPS) of the scale, which included physiologic and behavioral data.

Each patient was assigned a score for the RbtGS, the CPS and, consequently, for the composite pain scale. Score ranges were adjusted when evaluating Lop eared rabbit, since ear position was not assessable. Pain scores ranged from 0 to 24 for the CANCRS or from 0 to 22 for the Lop eared rabbit version of the CANCRS, from 0 to 14 for the CPS and from 0 to 10 for the RbtGS or from 0 to 8 for the Lop eared rabbit version of the RbtGS. In order to make the comparison between scales possible, for each scale, the scores were divided into four classes: no pain (NP), discomfort (D), moderate pain (MP), severe pain (SP); this allowed comparison with the four classes belonging to the PP (presumptive pain). Score ranges for the aforementioned classes are shown in Table 3.

**Table 3:** Ranges of the scores for each class of pain in CANCRS, RbtGS and CPS.

	NP	D	MP	SP
**CANCRS**	0–5	6–11	12–17	18–24
**RbtGS**	0–1	2–4	5–7	8–10
**CPS**	0–2	3–6	7–10	11–14

Scores were equally distributed in four pain classes. For CANCRS, scores from 0 to 5 were classified as NP, scores from 6 to 11 were classified as D, scores from 12 to 17 were classified as MP and scores from 18 to 24 were classified as SP.

For RbtGS, scores from 0 to 1 were classified as NP, scores from 2 to 4 were classified as D, scores from 5 to 7 were classified as MP and scores from 8 to 10 were classified as SP.

For CPS, scores from 0 to 2 were classified as NP, scores from 3 to 6 were classified as D, scores from 7 to 10 were classified as MP and scores from 11 to 14 were classified as SP.

Raters were asked to measure the time spent on each assessment, in order to define an average time needed to evaluate pain with the CANCRS, verifying if it would fit a clinical environment, where the clinician must identify pain as fast as possible.

### Statistical analysis

#### Inter-rater reliability

To assess inter-rater reliability, patients were evaluated independently, but at the same time, by two raters. Sixty-nine patients were assessed by a veterinarian and a student, whereas, forty-seven rabbits were assessed by two veterinarians. For both groups, intra-class correlation coefficient (ICC), involving a two-way random effect model with 95% confidence intervals, was calculated. Furthermore, Cohen’s kappa was determined for each parameter separately, considering sixty-nine rabbits assessed by a veterinarian and a veterinary medicine student, allowing an estimation of the inter-rater agreement. Ear position was assessed in a cohort of 56 rabbits, since 13 patients were Lop eared rabbits and ear position assessment is not possible in this breed. The results of inter-rater reliability were interpreted using Altman’s classification [29], in which values ranging from 0.81–1.0, 0.61–0.80, 0.41–0.60, 0.20–0.40 and <0.20 are considered very good, good, moderate, fair, and poor, respectively.

#### Validity

The accuracy of a tool in measuring a specific construct like pain, is construct validity [30]. Pain assessment was performed on each one of the 116 rabbits included in the study by two raters; therefore 232 evaluations were considered [30]. In this case, to test construct validity CANCRS, RbtGS and CPS. A chi squared test was used to verify how frequently the score assigned by using CANCRS, RbtGS and CPS had a correspondence with the PP class.

A p value ≤ 0.05 was considered significant, and a p value ≤ 0.001was considered extremely significant. Statistical analyses were performed with the software *R* version 3.2.2.

## Results

The mean time required to use the CANCR scale was 3.4 minutes (min 2.2—max 5.6 minutes).

### Inter-rater reliability

The ICC was 0.88 (p ≤ 0.001; 95% CI 0.81–0.92) between veterinarians (V) and students (S) and 0.94 (p ≤ 0.001; 95% CI 0.88–0.97) between V and V for the CANCRS, indicating very good inter-rater reliability.

The ICC was 0.97 (p ≤ 0.001; 95% CI 0.96–0.98) between V and S and 0.91 (p ≤ 0.001; 95% CI 0.83–0.95) between V and V for the CPS, indicating very good inter-rater reliability.

Finally, the ICC was 0.77 (p ≤ 0.001; 95% CI 0.66–0.85) between V and S and 0.88 (p ≤ 0.001; 95% CI 0.78–0.94) between V and V for the RbtGS, indicating good and very good inter-rater reliability respectively.

For the weighted Cohen’s kappa values, moderate agreement was obtained for cheek flattening (0.48; 95% CI 0.25–0.68), nostril shape (0.58; 95% CI 0.39–0.73) and whisker position (0.56; 95% CI 0.32–070). Good agreement was obtained for orbital tightening (0.68; 95% CI 0.43–0.76).

Ear position (0.78; 95% CI 0.68–0.97), pupil dilation (0.87; 95% CI 0.75–0.99), respiratory rate (0.97; 95% CI 0.90–1), respiratory pattern (0.89; 95% CI 0.74–1), heart rate (0.93; 95% CI 0.83–1), response to palpation (0.87; 95% 0.71–1), mental status (0.92; 95% 0.80–1) and vocalization (0.88; 95% CI 0.65–1) had very good inter-rater agreement. Cohen’s kappa coefficients are summarized in Table 4.

**Table 4:** Cohen’s kappa results between veterinarians and students for each parameter.

	Cohen’s kappa	CI
Orbital tightening	0.68	0.43–0.76
Cheek flattening	0.48	0.25–0.68
Nostril shape	0.58	0.39–0.73
Whisker position	0.56	0.32–0.70
Ear position	0.78	0.68–0.97
Pupil dilation	0.87	0.75–0.99
Respiratory rate	0.97	0.90–1
Respiratory pattern	0.89	0.74–1
Heart rate	0.93	0.83–1
Response to palpation	0.87	0.71–1
Mental status	0.92	0.80–1
Vocalization	0.88	0.65–1

### Validity

The four possible results matched PP classes: No Pain (NP), Discomfort (D), Moderate Pain (MP), and Severe Pain (SP).

The chi-squared test indicated that the CANCRS-scale and the RbtGS scores are distributed as expected according to P (P ≤ 0.05). Patients in a presumptive condition of pain absence (PP = NP) and rabbits in discomfort (PP = D) are mostly classified as NP or D; rabbits in a moderately pain condition (PP = MP) are mostly classified as D. None of the presumed cases of several pain (PP = SP) was detected by the CANCRS, although some cases were detected using the RbtGS. Otherwise, the test indicated that the CPS score frequencies are statistically different from the expected results based on PP (P > 0.05).

**Fig 1:**
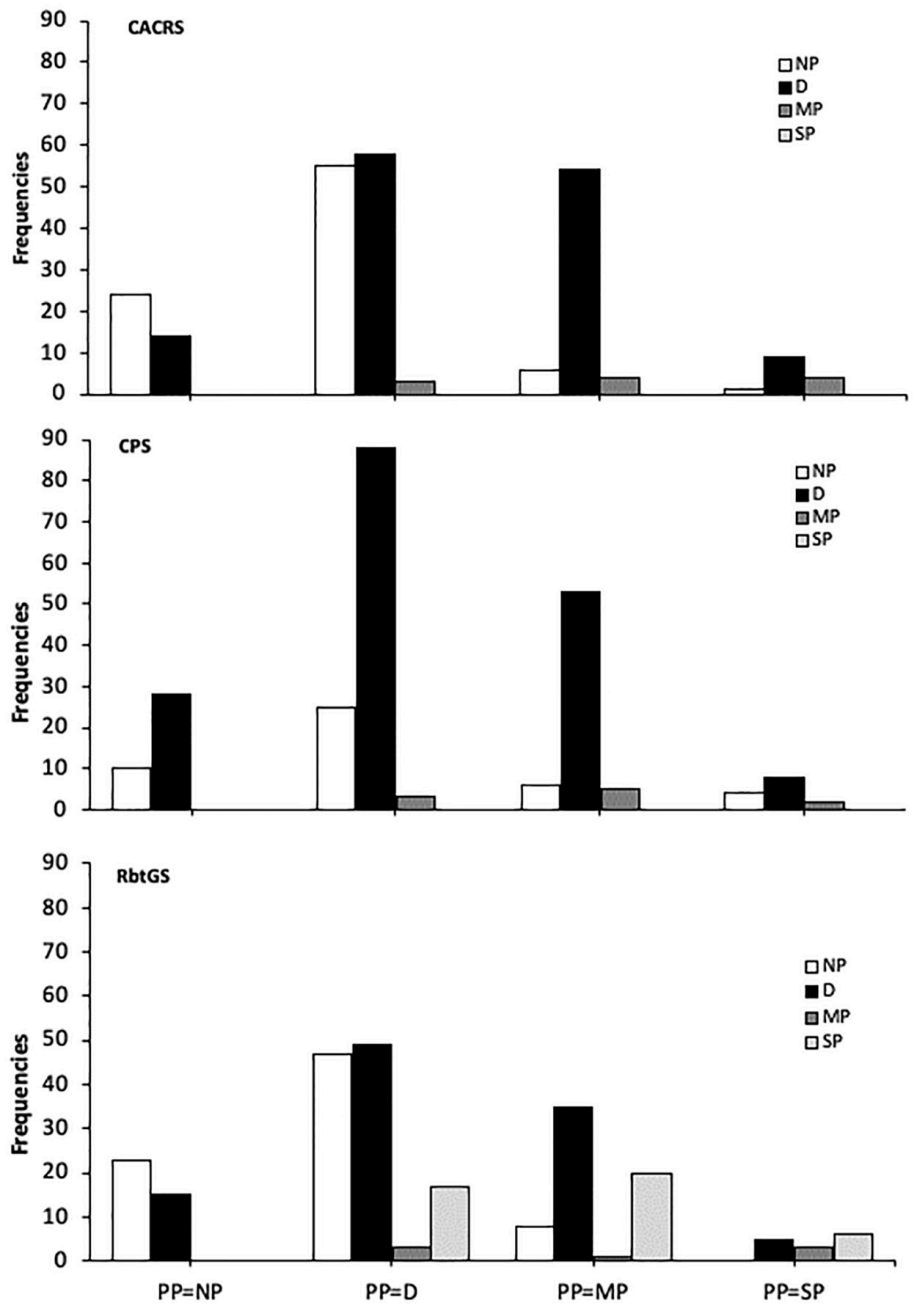
Distribution of the results obtained using CANCRS, CPS, and RbtGS related to presumptive pain classes (PP). CANCRS: results show that frequencies are not randomly obtained, but diagnosis obtained by assessing pain with the CANCRS are related to PP (p≤0.05). CPS: results show that frequencies could be randomly obtained, and that there is no relation between CPS and PP (p>0.005). RbtGS: results show that frequencies are not randomly obtained, but diagnosis obtained by assessing pain with the RbtGS are related to PP (p≤0.05).

Results for each scale are shown in Table 5 and Fig 1 summarizes how the results obtained with the three scales matched PP classes.

**Table 5:** Results for CANCRS, CPS and RbtGS compared to PP.

		PRESUMPTIVE PAIN (PP)			
		NP	D	MP	SP
CANCRS	NP	24 of 38	55 of 116	6 of 64	1 of 14
	D	14 of 38	58 of 116	54 of 64	9 of 14
	MP	0 of 38	3 of 116	4 of 64	4 of 14
	SP	0 of 38	0 of 116	0 of 64	0 of 14
CPS	NP	10 of 38	25 of 116	6 of 64	4 of 14
	D	28 of 38	88 of 116	53 of 64	8 of 14
	MP	0 of 38	3 of 116	5 of 64	2 of 14
	SP	0 of 38	0 of 116	0 of 64	0 of 14
RbtGS	NP	23 of 38	47 of 116	8 of 64	0 of 14
	D	15 of 38	49 of 116	35 of 64	5 of 14
	MP	0 of 38	3 of 116	1 of 64	3 of 14
	SP	0 of 38	17 of 116	20 of 64	6 of 14

A presumptive pain class (PP) was assigned to each patient at admission. Patients (n = 38) were classified as NP; patients (n = 116) were classified as D; patients (n = 64) were classified as MP; patients (n = 14) were classified as SP. Patients pain was then assessed. Results for CANCRS, CPS, RbtGS were divided for each pain class and listed in columns.

## Discussion

This study assessed the inter-rater reliability and construct validity of the CANCRS in a clinical environment. The scale is recommended for clinical use, for CANCRS construct validity was confirmed, and very good inter-rater reliability exists between veterinarians and veterinary medicine students. Results for RbtGS showed a good-to-very-good level of reliability, and the scale has construct validity. Although reliability results for CPS are very good for both veterinarians and veterinary medicine students, validity results show that using CPS alone is not effective, since presumptive pain classes and CPS results were differently distributed. The RbtGS is the only existing scale for acute pain evaluation in laboratory rabbits. It is based on five facial action units [15] that can be observed from a distance in a relatively short time, making the evaluation less stressful for the patient. RbtGS was developed on one rabbit breed only, NZW, since laboratory rabbits were supposed to be healthy and undergoing a routinely procedure such as ear tattooing [15]. The present study assessed the reliability and the construct validity of the RbtGS in a clinical setting, considering patients with several clinical conditions and testing the scale on various rabbit breeds and hybrids with many morphological variations.

RbtGS is included in the CANCRS; consequently, using it alone is faster. However, the clinical parameters that the latter scale adds to the RbtGS, should always be monitored during any clinical examination. Therefore, using the CANCRS can lead to a more comprehensive evaluation of the patient, as composite pain scales encourage the observer to consider some behavioral and physiological changes, that would not otherwise be evaluated, but their importance is undeniable as pain is a multidimensional experience [2]. However, it is important to bear in mind that none of the cases of presumed severe pain were detected by the CANCRS. This should be taken into consideration by the clinician when performing pain assessment and the recommendation is to always consider serious clinical conditions as cases of severe pain.

Currently, no gold standard for pain assessment in rabbits exists; therefore, results were compared to presumptive pain classes, according to what is reported in literature for other domestic mammals [2]. Although it should be remembered that these classes are only presumed, as pain is a subjective experience that is not possible to completely describe except for self-reported pain, and this is possibly only in human medicine [2]. Furthermore, pain intensity can change due to several factors, such as the concomitant presence of other sources of pain, previous painful experiences, the age and the gender of the animal [2,31]. These are significant limitations that should always be considered when dealing with pain assessment in veterinary medicine.

There are other limitations to bear in mind when applying physiological and behavioral parameters to evaluate pain. Physiological parameters can be altered in several circumstances such as stress, positive excitement and any pathological conditions [3]. Handling during the clinical examination is stressful for the patient, therefore physiological parameters such as respiratory and heart rates have only a limited function in pain assessment [7,10]; specifically heart rate is often hard to count, given high physiological frequencies typical of small mammals. Behavioral changes are considered to be a more reliable sign of pain and the return to a calm and unstressed behavior often coincides with pain resolution [32]. According to composite pain scales developed for other species [11,12,14], some behavioral changes are effective for pain assessment, but some of them may be hard to assess during a relatively short clinical examination (e.g. changes in grooming frequency) [30]. Although selected parameters do not represent a complete behavioral approach, acute behavioral changes during palpation and vocalizations could be a good indication of the pain the patient is experiencing. To properly assess the presence of abnormalities, familiarity with the typical behavior of the species and knowledge of the individual characteristics are fundamental [3]. The latter consideration encourages testing of the inter-rater agreement between veterinarians and veterinary medicine students for each parameter of the scale by assessing Cohen’s kappa. Results show that the agreement between raters for all parameters included in the CANCRS are good or very good. Specifically, the FAU included in the RbtGS results have a moderate inter-rater agreement, except for ear position, for which Cohen’s kappa result was very good. Although, we cannot exclude that this result is due to the small subject population considered. Strengths of this study include a relatively large sample size, compared to other papers [15,31], patients with a wide range of clinical conditions and morphological differences due to the variety of breeds and hybrids considered.

In conclusion, this study suggests that the CANCRS could be a useful tool in clinical practice in order to improve pain assessment.

Responsiveness of the scale of changes in pain level according to analgesic administration was not tested in the present study and internal validity represents another area for further study.

## Supporting information

S1 Dataset (XLSX)
